# Hospital characteristics and perioperative complications of Hispanic patients following reverse shoulder arthroplasty—a large database study

**DOI:** 10.1186/s42836-023-00206-2

**Published:** 2023-10-04

**Authors:** Nikit Venishetty, Garrett Sohn, Ivy Nguyen, Meesha Trivedi, Varatharaj Mounasamy, Senthil Sambandam

**Affiliations:** 1 Texas Tech University Health Sciences Center, El Paso, TX 5001 USA; 2 University of Texas Southwestern, Harry Hines Blvd, Dallas, TX 5323 USA; 3 University of Texas Southwestern, Dallas VAMC, Dallas, TX 4500 USA

**Keywords:** Perioperative complications, Reverse shoulder arthroplasty, Disparities in healthcare, Hispanic patients, Length of stay, Cost of care

## Abstract

**Background:**

Hispanic patients are the youngest and fastest-growing ethnic group in the USA. Many of these patients are increasingly met with orthopedic issues, often electing to undergo corrective procedures such as reverse shoulder arthroplasty (RSA). This patient population has unique medical needs and has been reported to have higher incidences of perioperative complications following major procedures. Unfortunately, there is a lack of information on the hospitalization data and perioperative complications in Hispanic patients following procedures such as RSA. This project aimed to query the Nationwide Inpatient Sample (NIS) database to assess patient hospitalization information, demographics, and the prevalence of perioperative complications among Hispanic patients who received RSA.

**Methods:**

Information from 2016–2019 was queried from the NIS database. Demographic information, incidences of perioperative complications, length of stay, and costs of care among Hispanic patients undergoing RSA were compared to non-Hispanic patients undergoing RSA. A subsequent propensity matching was conducted to consider preoperative comorbidities.

**Results:**

The query of NIS identified 59,916 patients who underwent RSA. Of this sample, 2,656 patients (4.4%) were identified to be Hispanic, while the remaining 57,260 patients (95.6%) were found to belong to other races (control). After propensity matching, Hispanic patients had a significantly longer LOS (median = 1.4 days) than the patients in the control group (median = 1.0,* P* < 0.001). The Hispanic patients (89,168.5 USD) had a significantly higher cost of care than those in the control group (67,396.1 USD, *P* < 0.001). In looking at postoperative complications, Hispanic patients had increased incidences of acute renal failure (Hispanics: 3.1%, control group: 1.1%, *P* = 0.03) and blood loss anemia (Hispanics: 12.7%, control group: 10.9%, *P* = 0.03).

**Conclusions:**

Hispanic patients had significantly longer lengths of stay, higher costs of care, and higher rates of perioperative complications compared to the control group. For patients who are Hispanic and undergoing RSA, this information will aid doctors in making comprehensive decisions regarding patient care and resource allocation.

## Introduction

Healthcare costs have continually grown over the past decade with over $4.3 trillion spent in 2021, accounting for 18.3% of the Gross Domestic Product [1]. Large joint arthroplasty surgeries have become a prominent focus of cost assessments due to their enormous volume and notable expenditure [2, 3]. Procedures such as reverse shoulder arthroplasty (RSA) have increasingly increased in popularity in the past decade for their effectiveness in pain relief and function-restoration. However, a large subset of the USA population remains hindered from accessing these procedures due to ongoing racial disparities and cost of procedures [4]. Moreover, minority groups such as Hispanics have been reported to have a higher expenditure on healthcare in comparison to white patients, which hinders patients from accessing major surgeries that are cost-intensive such as RSA [5]. Previous studies have shown that minorities have a much lower incidence of utilization of RSA as well [4, 6]. In addition, several studies have assessed factors that impact arthroplasty surgeries such as total hip arthroplasty (THA) and total knee arthroplasty (TKA), but there is a paucity of information on the complications of Hispanic patients following reverse shoulder arthroplasty [7–12].

It is crucial to note that Hispanics are the youngest and fastest-growing ethnic group in the USA, which may translate to a higher RSA burden in this population [13]. Unfortunately, Hispanics are met with worse health outcomes such as higher rates of obesity, lower rates of language fluency, and even high rates of the uninsured [14]. According to the 2020 Census Bureau, only 49.9% of Hispanics had private insurance coverage, compared to almost 73.9% of non-Hispanic whites [14]. Moreover, Hispanic populations have been documented to have worse outcomes after orthopedic procedures such as a total joint arthroplasty [5, 15, 16]. Therefore, it is crucial to understand the major perioperative complications and the associated hospital costs that occur within this population to inform healthcare providers with a more comprehensive medical assessment.

In this study, we used the Nationwide Inpatient Sample (NIS) Database to assess patient hospitalization information, demographics, and the prevalence of perioperative complications among Hispanic patients who received RSA. We theorize that Hispanic patients will have a longer length of stay (LOS), higher costs of care (COC), and significantly more perioperative complications.

## Methods

### NIS Database

The NIS database provides information on more than seven million hospital stays [17]. The large sample size allows for an examination of several ethnic groups such as Hispanics and the creation of national estimates. Over 49 states participate in the Healthcare Cost & Utilization project that is assessed by the NIS, and it comprises over 97% of the USA population [17]. Moreover, the NIS provides crucial information regarding hospitalization characteristics, demographics, and postoperative complications following major surgeries such as RSA. Our Institutional Review Board (IRB) exempted this project due to the publicly available de-identified information. Using data from 2016–2019, all patients having RSA-related ICD-10, the Tenth Revision, and Clinical Modification/Procedure Coding System (CMP) codes were incorporated into this project. The study sample was split into two groups: Hispanic patients and non-Hispanic patients (control).

Age, sex, ethnicity, and the existence of obesity were all considered while assessing demographic traits. Comorbidities, medical complications, and surgical complications were also described. In addition, home/routine, alternate facilities, and home healthcare (HHC) were examined as the patients’ post-hospitalization discharge destinations. The length of stay and care costs were also examined. Inclusion criteria were solely based on the ICD codes used in the NIS database. There were no exclusion criteria for the study as all variables were based on the ICD codes.

### Statistical evaluation

The statistical analyses were performed using IBM SPSS version 27.0 (IBM; Armonk, NY, USA). Patient demographic information was initially gathered through descriptive statistics. Numerical variables were assessed using *t*-tests, while chi-squared analysis was employed for binomial variables. Length of stay and total charges, continuous variables in this study, for both groups, were found not to follow a normal distribution. Thus, an independent sample Mann–Whitney U test was employed and the median and IQR were reported. On the other hand, the age for both groups followed a normal distribution. A matched analysis was used to adjust for the following comorbidity and preoperative variables: diabetes, age, gender, tobacco-related disorders, obesity, and elective vs non-elective admissions. When the incidence number was less than 5, Fischer exact testing was employed. A significance level of *P* < 0.05 was set for all tests. The odds ratios and their corresponding 95% confidence intervals were calculated by comparing the incidence ratio between the Hispanic group and the non-Hispanic group.

## Results

### Patient demographic data

A total of 59,916 patients were identified in the NIS database who underwent RSA during the study period of 2016–2019. Of this sample, 2,656 patients (4.4%) were identified to be of Hispanic ethnicity, while the remaining 57,260 patients (95.6%) were found to be of other ethnic origins (control). Hispanic patients on average were significantly younger (70.6 years) than the control group (71.4 years, *P* < 0.001). There was a significantly greater proportion of females in the Hispanic group compared to the control group (Table 1).

**Table 1:** Patient demographic characteristics of Hispanics and Non-Hispanics

**Variables**	**Hispanics (*n* = 2,656)**	**Non-Hispanics (*n* = 57,260)**	**Significance**
Age (years)	70.6 (SD = 9.2)	71.4 (SD = 8.6)	** < 0.001**
Female	1,778 (66.9%)	34,514 (60.2%)	** < 0.001**
Obesity	520 (19.7%)	11,444 (19.9%)	0.28
Race			
Caucasian	N/A	50,942 (88.9%)	N/A
African American	N/A	2,542 (4.4%)	N/A
Hispanic	2,656 (100%)	N/A	N/A
Asian	N/A	341 (0.6%)	N/A
Native American	N/A	206 (0.3%)	N/A
Other	N/A	881 (1.5%)	N/A

Bolded values indicate statistical significance

### Unmatched analyses: patient admission characteristics

Hispanic patients had a significantly longer LOS (median = 1.4 days, IQR: 1, 2) than the non-Hispanic group (median = 1.0 days, IQR: 1, 2) (*P* < 0.001). The Hispanic patients (median = 89,168.5 USD, IQR: 61,720.5, 125,569.0) had a higher COC than their non-Hispanic counterparts (median = 66,602.1 USD, IQR: 49,419.3, 93,568.8) (*P* < 0.001). A significantly larger proportion of patients in the Hispanic group had diabetes with and without complications than those in the control group (Table 2).

**Table 2:** Admission characteristics of Hispanic patients and the controls

**Variables**	**Hispanics (*n* = 2,656)**	**Non-Hispanics (*n* = 57,260)**	**Significance**
Length of Stay (Days)(Median)^b^	1.4 (IQR: 1, 2)	1.0 (IQR:1, 2)	** < 0.001**
Total Charges (USD) (Median)^a^	89,168.5 (IQR: 61,720.5, 125,569.0)	66,602.1 (IQR: 49,419.3, 93,568.8)	** < 0.001**
Tobacco-Related Disorder	285 (10.7%)	9,359 (16.3%)	** < 0.001**
Diabetes without Complications	584 (21.9%)	8,073 (14.1%)	** < 0.001**
Diabetes with Complications	19 (0.7%)	99 (0.1%)	** < 0.001**
Primary Expected Payer			
Medicare	1,879 (70.7%)	43,940 (76.7%)	** < 0.001**
Medicaid	175 (6.6%)	1,483 (2.6%)	** < 0.001**
Private Insurance	396 (14.9%)	9,121 (15.9%)	** < 0.001**
Self-Pay	32 (1.2%)	240 (0.4%)	** < 0.001**
No Charge	(0.1%)	17 (0.01%)	**0.03**
Other	178 (6.7%)	2423 (4.2%)	** < 0.001**
Patient Discharge			
Home/Routine	1,696 (63.8%)	37,654 (65.7%)	** < 0.001**
Another Type of Facility	340 (12.8%)	7,826 (13.7%)	0.13
Home Healthcare (HHC)	624 (23.5%)	11,589 (20.3%)	** < 0.001**

Bolded values indicate statistical significance. As per the Healthcare Cost and Utilization Project data agreement, numbers ranging from 1 to 10 were not reported

^a^Due to an incidence of 0 in one of the groups, it was not possible to calculate the odds ratio and significance values

^b^Length of stay and Total charges were found not to follow a normal distribution, thus an Independent-Samples Mann–Whitney U test was performed

In looking at the patient’s primary expected payer, Hispanic patients had significantly higher use of Medicaid (6.6%) than the non-Hispanic group (2.6%, *P* < 0.001). In addition, a larger proportion of Hispanic patients (1.2%) utilized self-pay as compared to the non-Hispanic group (0.4%, *P* < 0.001). On the other hand, non-Hispanic patients (control) had a significantly higher incidence of utilizing Medicare and private insurance in comparison to Hispanic patients (*P* < 0.001).

There was a significantly lower number of Hispanic patients that were discharged home (63.8%) in comparison to the control group (65.7%, *P* < 0.001). However, a significantly larger proportion of Hispanic patients utilized home healthcare (23.5%), as compared to patients in the control group (20.3%, *P* < 0.001) (Table 2).

### Unmatched analyses: perioperative complications

In analyses of perioperative complications following RSA, the Hispanic group had a significantly higher incidence of acute renal failure, blood loss anemia, and blood transfusion. The Hispanic group had a higher incidence of developing acute renal failure (3.1%) than patients in the non-Hispanic group (2.1%, *P* < 0.001). Moreover, Hispanic patients were more likely to develop blood loss anemia than patients in the control group (12.7% vs. 10.1%, *P* < 0.001). Lastly, Hispanic patients had a higher incidence of blood transfusions (2.6%) than those in the control group (1.9%, *P* = 0.02). Postoperative complications such as pneumonia, pulmonary embolism, periprosthetic fracture, periprosthetic infection, periprosthetic dislocations, and wound dehiscence were similar in both groups (Table 3).

**Table 3:** Unmatched analysis: perioperative complications of Hispanics and non-Hispanics

**Post Operative Variables**	**Hispanic (*n* = 2,656)**	**Non-Hispanic (*n* = 57,260)**	**Odds Ratio (Hispanic/Control Group)**	**Odds Ratio 95% Confidence Interval**	**Significance**
Died During Hospitalization	(0.001%)	38 (0.01%)	0.56	(0.08, 4.11)	0.47
Acute Renal Failure	83 (3.1%)	1,251 (2.1%)	1.43	(1.15, 1.80)	** < 0.001**
Myocardial Infarction	0%	29 (0.01%)	*	*	*
Blood Loss Anemia	336 (12.7%)	5,800 (10.1%)	1.28	(1.14, 1.44)	** < 0.001**
Pneumonia	10 (0.4%	217 (0.3%)	0.99	(0.53, 1.87)	0.57
Blood Transfusion	68 (2.6%)	1,109 (1.9%)	1.32	(1.04, 1.70)	**0.02**
Pulmonary Embolism	(0.001%)	73 (0.01%)	0.58	(0.14, 2.39)	0.34
Deep Vein Thrombosis	(0.001%)	45 (0.01%)	1.43	(0.45, 4.61)	0.36
Periprosthetic Fracture	(0.001%)	129 (0.02%)	0.66	(0.25 1.80)	0.29
Periprosthetic Dislocation	35 (1.3%)	965 (1.7%)	0.78	(0.74. 3.31)	0.08
Periprosthetic Infection	17 (0.6%)	328 (0.6%)	1.11	(0.68, 1.81)	0.38
Periprosthetic Mechanical Complication	18 (0.7%)	734 (1.3%)	0.52	(0.33, 0.84)	**0.004**
Superficial SSI	0%	0.001%	*	*	*
Wound Dehiscence	(0.001%)	18 (0.003%)	1.19	(0.16, 8.95)	0.58

Bolded values indicate statistical significance. As per the Healthcare Cost and Utilization Project data agreement, numbers ranging from 1 to 10 were not reported

^*^Due to an incidence of 0 in one of the groups, it was not possible to calculate the odds ratio and significance values

### Matched analyses: hospital characteristics

After propensity matching, Hispanic patients continued to have a longer LOS (median = 1.4 days, IQR: 1, 2) than their non-Hispanic counterparts (median = 1.0 days, IQR: 1, 2) (*P* < 0.001). In addition, the cost of care was higher for Hispanic patients (median = 89,168.5 USD, IQR: 61,720.5, 125,569.0) than for the non-Hispanic patients (median = 67,306.1 USD, IQR:50,262.5, 96,674.0) (*P* < 0.001). These results are shown in Table 4.

**Table 4:** Matched analysis: hospital characteristics and perioperative complications of Hispanics and non-Hispanics

**Post Operative Variables**	**Hispanics (*n* = 2,656)**	**Non-Hispanic (*n* = 2,662)**	**Odds Ratio (Hispanic/Control Group)**	**Odds Ratio 95% Confidence Interval**	**Significance**
Length of Stay (Days) (Median)**	1.4 (IQR: 1, 2)	1 (IQR: 1, 2)	N/A	N/A	< 0.001
Total Charges (USD) (Median)**	89,168.5 (IQR: 61,720.5, 125,569.0)	67,306.1 (IQR:50,262.5, 96,674.0)	N/A	N/A	< 0.001
Died During Hospitalization	(0.001%)	4 (0.1%)	0.25	(0.03, 2.23)	0.18
Acute Renal Failure	83 (3.1%)	29 (1.1%)	1.23	(1.21, 1.56)	**0.03**
Myocardial Infarction	0%	2 (0.00%)	*	*	*
Blood Loss Anemia	336 (12.7%)	290 (10.9%)	1.18	(1.01, 1.40)	**0.03**
Pneumonia	10 (0.4%	11 (0.4%)	0.91	(0.39, 2.14)	0.49
Blood Transfusion	68 (2.6%)	70 (2.6%)	0.97	(0.69, 1.36)	**0.46**
Pulmonary Embolism	(0.001%)	3 (0.02%)	0.66	(0.11, 3.99)	0.49
Deep Vein Thrombosis	(0.001%)	2 (0.01%)	1.49	(0.25, 8.98)	0.50
Periprosthetic Fracture	(0.001%)	6 (0.2%)	0.67	(0.18, 2.36)	0.38
Periprosthetic Dislocation	35 (1.3%)	55 (2.1%)	0.63	(0.41. 0.97)	0.02
Periprosthetic Infection	17 (0.6%)	15 (0.56%)	1.13	(0.57, 2.27)	0.43
Periprosthetic Mechanical Complication	18 (0.7%)	40 (1.5%)	0.59	(0.33, 0.96)	**0.04**
Superficial SSI	0%	0%	*	*	*
Wound Dehiscence	(0.001%)	0%	*	*	*

Bolded values indicate statistical significance. As per the Healthcare Cost and Utilization Project data agreement, numbers ranging from 1 to 10 were not reported

^*^Due to an incidence of 0 in one of the groups, it was not possible to calculate the odds ratio and significance values

^**^Length of stay and Total charges were found not to follow a normal distribution, thus an Independent-Samples Mann–Whitney U test was performed

### Matched analyses: perioperative complications

After a 1:1 propensity matching, the Hispanic patients remained to have significantly higher incidences of acute renal failure, blood loss anemia, and periprosthetic mechanical complications. However, the significance of blood transfusions between the two groups attenuated. Hispanic patients had a much higher incidence of developing acute renal failure (3.1%) than patients in the non-Hispanic group (1.1%, *P* = 0.03). In addition, Hispanic patients were more likely to develop blood loss anemia than patients in the control group (12.7% vs. 10.9%, *P* = 0.03). Postoperative complications such as pneumonia, pulmonary embolism, periprosthetic fracture, blood loss transfusion, periprosthetic infection, periprosthetic dislocations, and wound dehiscence were similar in both groups (Table 4).

## Discussion

With the growing prevalence of reverse shoulder arthroplasty in the USA, Hispanic patients are utilizing the procedure increasingly [2, 18]. However, limited information is available regarding the postoperative complications of this procedure. Unfortunately, Hispanics have reportedly a significantly higher number of postoperative complications following major surgeries [5, 15, 16, 19]. Hispanics encounter numerous obstacles to the access to high-quality healthcare, including difficulty obtaining health insurance and a lack of cultural and linguistic compatibility with healthcare practitioners, as previous research has shown [20]. The higher number of complications results from disparities in surgical management and health care delivery and necessitates studies to provide information to improve health outcomes for Hispanic populations after major surgeries such as RSA.

In our study, even after propensity matching, Hispanic patients were found to have a significantly longer length of stay and cost of care in comparison to non-Hispanic patients. Ghosh et al. [21] found no significant difference in LOS between Hispanic patients and White patients, and only Black patients had significantly longer LOS. To our knowledge, RSA in Hispanics has not been assessed before. However, previous studies have shown that Hispanics have longer LOS after major surgeries, such as total hip arthroplasty and other medical complications, such as hospitalization for heart failure [9, 22]. Moreover, the longer LOS has been reported to contribute to a higher COC following procedures such as total shoulder arthroplasty and RSA [23, 24]. Potential conclusions for this difference in LOS might stem from variable communication with patients about their discharge plans. This can be demonstrated in the fact that non-Hispanic patients were more likely to be discharged home in comparison to Hispanic patients. Another potential cause may be the scarcity of case management support for effective transitions of care [22]. Zheng et al. proposed that hospitals with established relationships with facilities that take in under-served patients should focus their time and resources on that area [22]. Moreover, additional financial incentives may be proposed to encourage facilities to accept uninsured and under-served patients such as Hispanics.

Apart from longer LOS, we also found that Hispanic patients had a significantly higher COC than non-Hispanic whites. Sleiman et al. [24] found that lower-income patients had a higher COC in comparison to higher-income patients after RSA; however, they did not stratify their study population by ethnicity. Similar to our results, previous studies have reported that Hispanics had a larger hospital expenditure, but these studies did not look at Hispanics after large operations such as total joint arthroplasty [22, 25, 26]. It is speculated that the lack of insurance for Hispanics results in a higher cost of care, as we found that Hispanic patients were significantly less likely to have private insurance and Medicare coverage. In addition, Hispanic patients in our study were less likely to be discharged home after undergoing RSA. Not only does this contribute to the longer length of stays reported, but also may play a critical role in the rising expenses that this population encounters.

Operative complications after major procedures such as RSA can precipitate severe postoperative complications such as bleeding, wound complications, thromboembolic disease, infections, and periprosthetic fractures. However, to the best of our knowledge, no study has previously assessed post-RAS complications in Hispanics. Our unmatched analysis revealed that Hispanic patients were more likely to require blood transfusions and the matched analysis showed that they tended to suffer from blood loss anemia following RSA, in comparison to the controls. A study by Qian et al. [27] found that minority patients were 1.5 times more likely to necessitate a red blood cell transfusion during major surgeries such as coronary artery bypass surgery and total hip replacement. Minority patients such as Hispanics may need more blood transfusions due to worse surgical techniques used, which results in more blood loss as compared to white patients [27, 28]. Other studies have found that black patients are associated with higher rates of blood transfusion during surgery [27, 29]. However, these studies did not look at Hispanic populations, but it is important to note that minority populations are met with higher needs for blood transfusion after major surgeries. In contrast, previous studies have shown that minority groups, including Hispanics, are less likely to obtain blood transfusions in comparison to their white counterparts [30]. Our literature search found very limited studies that looked at blood transfusions in Hispanic populations. Therefore, we believe this study provides a new perspective on how providers should treat minority populations such as Hispanics during major surgeries like RSA.

Lastly, we found that Hispanics were significantly more likely to develop acute renal failure following RSA. Although Hispanics are documented to have higher rates of chronic kidney disease and acute renal failure than non-Hispanic whites (almost 1.3 times more likely to develop), there is a scarcity of information on the development of acute renal failure in this population after total joint arthroplasty [31, 32]. In addition, the higher rates of acute renal failure may contribute to the higher rates of blood loss anemia found in Hispanics. Studies have found that chronic kidney disease patients are more prone to blood loss anemia necessitating blood transfusion due to multiple factors including preoperative anemia of chronic disease, low erythropoietin level, and perioperative higher bleeding risk due to uremic effect on the platelet [33–35]. Thus, healthcare providers should be wary of this complication when dealing with this population by being prepared with iron supplementation and potential red blood cell transfusion.

## Limitations

We acknowledge that the NIS (National Inpatient Sample), the most comprehensive available database encompassing all participants, may have certain limitations in accurately capturing long-term outcomes of reverse shoulder arthroplasty in Hispanics. While the NIS data are generally precise with a specificity of over 92%, there can be occasional gaps or deficiencies in the data.

Moreover, the validity of the findings could be compromised when relying on ICD codes to compare patient samples. A more accurate approach would involve comparing precise data from individual institutions, as this would provide a more comprehensive representation of characteristics of the patient groups. Despite these considerations, it’s important to highlight that this study, with its large sample size, provides valuable insights into the characteristics of Hispanic patients undergoing RSA.

## Conclusion

To our knowledge, this study represents the first research effort to examine the perioperative complications and hospital admission characteristics among Hispanic individuals undergoing reverse shoulder arthroplasty. Hispanic patients have longer LOS, higher costs of care, fewer insurance rates, lower home discharge and more severe postoperative complications following RSA. The aim of this study is to offer valuable information regarding the perioperative outcomes of RSA specifically in Hispanic patients. The findings of this study hold significance for providers, healthcare organizations, and clinicians involved in the care of Hispanic patients. Moreover, when assessing RSA indications for Hispanic populations, it is important for providers to consider these patients for overnight admission in higher-level centers and follow-up care centers instead of outpatient surgery or ambulatory surgery centers. Additionally, it is essential to consider the higher incidence of perioperative complications observed within this patient group when evaluating hospital expenditures. The data gathered from this study will assist healthcare providers in making informed decisions regarding patient care and resource allocation specifically for Hispanic individuals undergoing RSA.

## Data Availability

The datasets used and/or analyzed during the current study are available from the corresponding author upon reasonable request.
